# Exploring the effect of femtosecond laser surface treatment on the bond strength of zirconia to metal brackets: an in vitro study

**DOI:** 10.1186/s12903-025-06996-y

**Published:** 2025-10-21

**Authors:** Lobna Shalaby, Hatem Saifeldin, Fatma Abdel Samad, Tarek Mohamed, Yosra El Demery

**Affiliations:** 1 Lecturer of Orthodontics, Orthodontic Department, Faculty of Dentistry, Ain Shams University, Organization of African Unity, 11566 Cairo, Egypt; 2 Associate Professor of Orthodontics, Orthodontic Department, Faculty of Dentistry, Ain Shams University, Organization of African Unity, 11566 Cairo, Egypt; 3 Teaching Assistant of Laser Physics, Laser Institute for Research and Application (LIRA), Beni-Suef University, 62511 Beni-Suef, Egypt; 4 Professor of Laser physics, Laser Institute for Research and Applications (LIRA), Beni-Suef University, 62511 Beni-Suef, Egypt; 5 Researcher, Fixed and Removable Prosthodontics Department, Oral and Dental Research Institute, National Research Center, 12622 Cairo, Egypt

**Keywords:** Femtosecond laser, Zirconia, Orthodontic brackets, Shear bond strength, Surface treatment

## Abstract

**Objective:**

To investigate the effect of zirconia (Zr) surface treatment with different femtosecond laser (FS) wavelengths on the shear bond strength (SBS) of metal brackets to multilayer zirconia.

**Methods:**

A total of sixty-four zirconia disc-shaped samples were randomly divided into four equal groups (*n* = 16). Airborne- particle abrasion (APA) utilizing 50 μm alumina particles (Al_2_O_3_) was applied to the first group, while the other three groups were treated with FS laser light at wavelengths of 385 nm, 800 nm, and 870 nm, respectively. Orthodontic metal brackets were bonded at the center of zirconia samples using universal bond and light-cured resin cement. Specimens were tested for SBS with a universal testing machine in occlusogingival direction at a crosshead speed of 0.5 mm/min until fracture. The adhesive remnant index (ARI) was registered, and surfaces were observed under stereomicroscope. Scanning electron microscopy (SEM) was utilized to analyze and compare the morphology of treated and untreated zirconia surface samples.

**Results:**

The APA group showed the greatest SBS value (17.23 ± 2.02 MPa), with no significant difference from the FS with 800 nm group (15.07 ± 3.42 MPa) or the FS with 870 nm group (13.01 ± 2.74 MPa). The FS 385 nm group showed significantly the lowest SBS value (8.07 ± 1.26 MPa) with *P* = 0.0001.

**Conclusions:**

The femtosecond laser irradiation at 800 nm and 870 nm achieved comparable shear bond strength (SBS) to airborne-particle abrasion (APA), indicating its potential as an alternative zirconia conditioning method, although it did not surpass APA. Its effectiveness was highly dependent on the selected wavelength parameters.

## Introduction

 The demand for adult orthodontic treatment has shown significant increase in contemporary orthodontic practice. Nowadays, extensive anterior and posterior restorations in the form of crowns or laminate restorations are frequently encountered. The necessity of bonding brackets to different fixed restorations became an unavoidable process in everyday practice [1, 2].

Ceramic restorations with their different types including feldsphatic porcelain, lithium disilicate-reinforced glass ceramics, and zirconia are widely used in current dental practice [3]. Zirconia restorations are widely used due to their high strength, fracture resistance, and biocompatibility, but they are not the most esthetic ceramic available; lithium disilicate glass ceramics typically offer superior translucency and color matching [4, 5].

Ceramic restorations can present considerable challenge for orthodontists, as establishing adequate bond strength on ceramic surfaces is difficult due to the glazed layer which interfere with the adhesion process [3, 6]. To improve the bond strength of orthodontic attachments to such restorations, various conditioning techniques have been developed in the form of mechanical, chemical, or a combination of both, aimed at modifying the ceramic properties and enhancing bond strength [7]. Mechanical approaches, such as airborne-particle abrasion with aluminum oxide, diamond bur application, and laser irradiation, are employed to create micromechanical retentions. On the other hand, chemical methods aim to produce a porous ceramic surface, commonly utilizing agents like phosphoric acid (PhA), and hydrofluoric acid (HF), silane [3, 7–10].

Following different surface treatments of ceramic restorations, various adhesive primers have been developed to facilitates chemical bonding between the organic groups of resin-based adhesives and the substrate surface [7]. Recently, “universal primers” have been introduced for broader application on both metals and ceramics, including zirconia and glass ceramics. Unlike specific primers, these materials incorporate silane and multiple functional monomers, such as 10-methacryloyloxydecyl dihydrogen phosphate (10-MDP), allowing chemical bonding with a wide range of restorative substrates [10]. Finally, the last step of bonding is the placement of the bonding resin on bracket base and light curing.

Bonding orthodontic brackets to zirconia restorations is a challengeable procedure because conventional acid etching is ineffective due to the absence of silica phase and the vitreous silane phase [11–13]. Establishing a reliable bonding protocol is essential to ensure durable and efficient adhesion that meets the requirements of orthodontic treatments. The main purpose of zirconia surface treatments is to increase bond strength, with improved wettability being a secondary effect.

For silica-based ceramics, such as lithium disilicate-reinforced glass ceramics, the standard treatment is hydrofluoric acid (HF) etching followed by silanization [14, 15]. However, in case of zirconia restorations, hydrofluoric acid etching and silane treatment are not effective [11, 12, 14, 15]. Airborne-particle abrasion (APA) with alumina particles, improves bonding to zirconia ceramics through creating micro-irregularities and increasing surface roughness [11]. Many studies have claimed increased effectiveness of this approach making it a preferred method in clinical practice [11–13, 16]. APA does not involve heat generation, however some studies reported unfavorable effects claiming that the mechanical impact of those particles with Zr surfaces might sometimes cause microcracks or surface damage which may potentially compromise the long-term performance of those restorations under stressful oral cavity conditions [17, 18].

Laser treatments have emerged as an alternative to traditional surface conditioning methods to enhance bonding between zirconia and resin cements, many of them found promising results [19, 20]. Different types of lasers, including CO_2_, Er: YAG, and Nd: YAG, have been investigated for their ability to modify ceramic surfaces and enhance bonding with promising outcomes [11, 21]. Kumar et al. concluded that lasers could offer an alternative surface treatment method due to their capability of reducing the negative impact of grit-blasting on the aesthetic and mechanical properties of zirconia [22]. Nevertheless, some studies reported adverse effect in the form of microcracks on ceramic surfaces with Nd: YAG and CO_2_ laser due to thermal damage might negatively affect the material’s fracture toughness [23, 24].

Recently, femtosecond (FS) laser gained attention as a novel method in dentistry due to its ability to emit ultrashort pulses that minimize thermal damage and provide precise surface modifications [25]. The FS Ti: Sapphire laser emits pulses in the femtosecond range (1 fs = 10^−15^ s), which significantly reduces heat transfer to the material, thus minimizing the risk of thermal damage compared to other laser types [26, 27]. This precision makes FS lasers an attractive option for dental applications, as they can create micro-retentive features on zirconia surfaces without compromising the material’s integrity [25].

Previous studies have demonstrated the potential of FS laser as a ceramic surface conditioning procedure to enhance bond strength to dental materials such as enamel, dentin, and different ceramics [28–31]. Nevertheless, research specifically focusing on the ideal pretreatment protocol for zirconia ceramics and the optimal FS laser settings for metal bracket bonding has been limited. Erdur et al. performed their study on feldspathic and e-Max ceramics not Zirconia ceramics [31]. Other studies were performed on zirconia ceramics but explored different parameter for femtosecond laser settings. García-Sanz et al. investigated the effect of femtosecond laser irradiation, both alone and in combination with sandblasting, on the shear bond strength (SBS) of metallic and ceramic brackets to zirconia, and compared the results with other conditioning techniques such as sandblasting, silica coating, and silane application [32]. Two years later, García-Sanz et al. performed another study focusing on FS irradiation surface treatment, testing combinations of different output power (200 mW and 300 mW), and inter-groove distance, but the a pulse width (30 fs), wavelength (800 nm), and a repetition rate (1 kHz for 12 min) settings were fixed for all groups [33]. Yucel et al. evaluated the effect of different femtosecond laser beam angles (30 and 90°) and the effect of three different shapes of the formed surfaces (spiral, square, and circular), on the roughness and shear bond strength (SBS) of resin cement to zirconia ceramic [34].

To date, few studies have investigated the influence of various femtosecond laser settings on the SBS of brackets and resin cements to zirconia [32–35], but non of them have addressed the effect of varying femtosecond laser wavelength settings on the shear bond strength (SBS) of brackets bonded to zirconia. Thus, the purpose of this in vitro investigation was to assess the shear bond strength of metal brackets to multilayer zirconia after femtosecond laser surface treatments with varying wavelength parameters and to determine the mode of bond failure associated with each treatment protocol. The null hypothesis assumed that variations in femtosecond laser wavelength parameters would have no effect on the shear bond strength between metal brackets and zirconia surfaces.

## Materials and methods

### Ethical approval and sample preparation

The study was exempted from review because it is a laboratory study not involving the use of any animal or human data. An exemption number was submitted by the by the Institutional Ethical Committee, Faculty of Dentistry, Ain Shams University (FDASU-Rec ER012362). The sample size was calculated based on the previous study performed by García-Sanz et al. [33]. According to this study, the minimally accepted sample size was sixteen per group, when mean standard deviation of group I was 5.92 ± 1.12 while estimated mean difference was 6, when the power was 80% & type I error probability was 0.05. The t test was performed by using P.S. power 3.1.6.

**Fig. 1:**
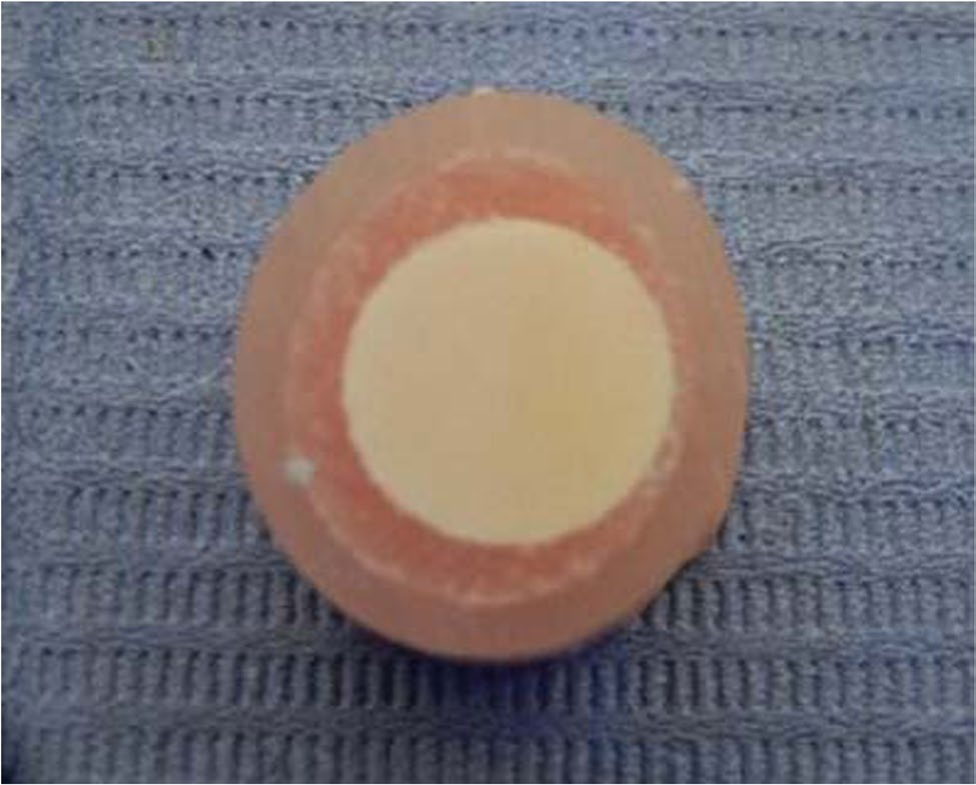
Zirconia discs mounted in acrylic blocks

Sixty-four zirconia samples were fabricated from ultra-translucent zirconia (5Y-YSZ Katana UTML, Kuraray Noritake, Japan). Disc-shaped specimens were prepared from a blank using a low-speed saw (IsoMet 4000, Buehler, USA) under water irrigation with dimensions 14 mm in diameter and 2 mm thickness. These discs were mounted in acrylic blocks to facilitate their manipulation and handling during different experimental procedures (Fig. 1).

### Experimental groups and procedures

All specimens were polished with silicon carbide paper (600-grit) to standardize the zirconia surface roughness before any surface treatment, then ultrasonically cleaned with pure ethyl alcohol for 15 min, dried with a mild air stream, and divided into four equal groups (*n* = 16) based on the surface treatment protocols tested.

In group I, the multilayer zirconia surfaces were treated by APA and considered as the gold standard reference group. The APA was performed using alumina particles (Al_2_O_3_) with a mean particle size of 50 μm, pressure of 4 bars, at 10 mm distance from the nozzle of the air particle abrasion machine (Renfert, Basic Eco, Germany) [36, 37]. The nozzle was held at an incident angle of 90ᴼ for 20 s. In groups II, III, and IV, the multilayer zirconia surfaces were treated with FS laser light with wavelengths of 385 nm, 800 nm, and 870 nm respectively.

### Femtosecond irradiation setup

The FS laser light pulses were delivered by the fully tunable laser system (INSPIRE HF100) from Spectra-Physics, which was pumped by a mode-locked femtosecond Ti: Sapphire laser (MAI TAI HP) from Spectra-Physics with an average power of 1.5–2.9 W and a repetition rate of 80 MHz. The INSPIRE HF100 laser system was operated by the fundamental IR pump wavelengths and nonlinear optics modes of operation. These modes of operation allowed the output wavelength range between 345 nm and 2500 nm [38].

**Fig. 2:**
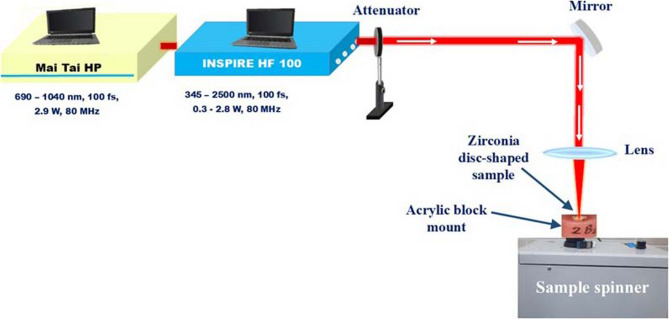
Experimental schematic diagram for irradiating Zirconia surfaces with femtosecond laser using different laser wavelengths

In groups II, III, and IV zirconia samples were ablated using a FS laser light with different excitation wavelengths of 385 nm, 800 nm, and 870 nm respectively, at a constant average power of 300 mW, and an exposure time of 20 min as presented in the schematic diagram shown in Fig. 2. The laser power was controlled and adjusted as required using a power attenuator. The incoming laser beam was passed through an iris diaphragm with a diameter of 8 mm at the 1/e^2^ width. The beam was focused to beam waist with a diameter of 30 μm onto the sample surface using a plano-convex lens with a focal length of 50 mm. The obtained laser fluence values F were calculated via formula: F = 2 × E/(π × ω_0_^2^), where E is energy per pulse and ω_0_ is beam radius [39]. The zirconia discs were mounted on a motorized sample spinner, rotating in a circular path perpendicular to the incident laser beam. Circular patterns were produced on the zirconia surfaces at radii of 5 mm, 3 mm, and 1 mm, with corresponding exposure times of 20, 12, and 4 min, respectively. The fabrication process involved maintaining a fixed scan speed of 177 rounds per minute (RPM).

### Bracket bonding procedure

A single lower incisor orthodontic metal bracket (Orthopro, USA) measuring 3 × 4 mm was bonded at the center of each specimen using universal bond (Tetric N-Bond Universal, Ivoclar, Vivadent) and light cure adhesive paste (Transbond XT, 3 M Unitek) according to the manufacturer instructions. Bonding procedure was performed by the same operator for standardization. The bracket zirconia samples were exposed to a light curing system (Mini S, RTA, Woodpecher) at 500 mW/cm^2^ intensity for 20 s, aimed at the gingival and occlusal bracket edges, to polymerize both the bond and the adhesive layer. The samples were kept for 24 h at 37°C in distilled water.

### Shear bond strength (SBS) test

**Fig. 3:**
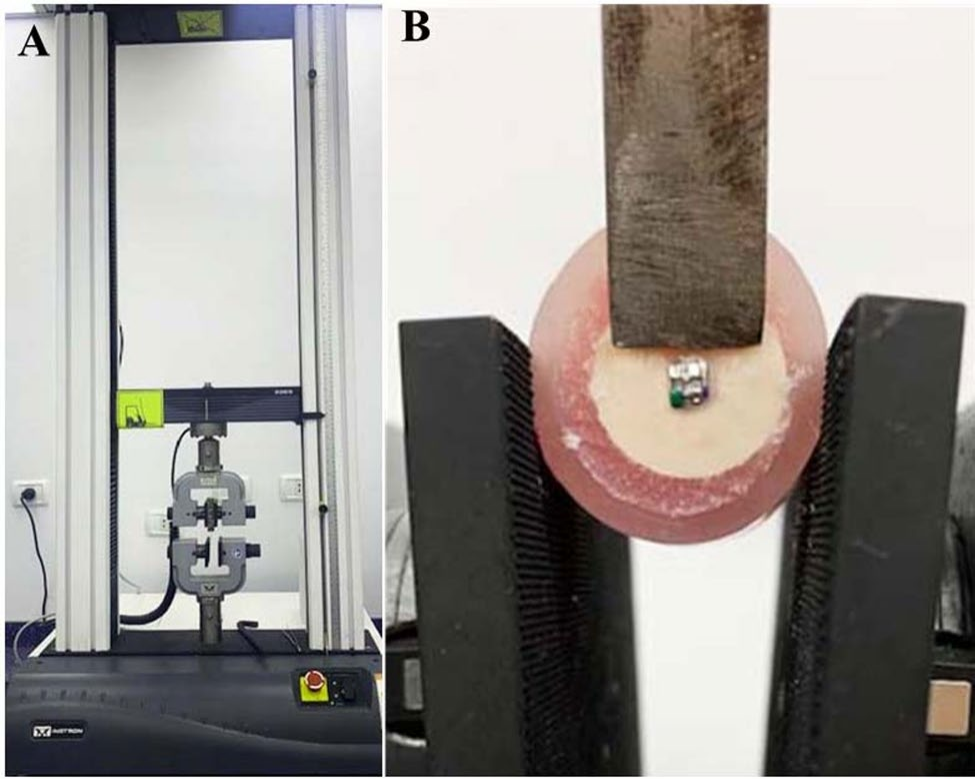
**A** Instron universal testing machine. **B** SBS testing process using a mono-beveled chisel held in a parallel direction to Zirconia discs to apply force on samples in an occlusogingival direction

The SBS was measured using universal testing machine (Instron 3365, Instron Corporation, England) with the mono-beveled chisel attached to the upper movable compartment of the testing machine to apply a compressive loading on each specimen (Fig. 3A). The load was applied in the occlusogingival direction at a crosshead speed of 0.5 mm/min. The chisel tip was settled to touch only the bracket base (Fig. 3B). The maximum failure load was recorded in Newton (n). The maximum failure load was then divided by the bracket base surface area, measured using a digital caliper, to present the bond strength in MPa.

### Adhesive remnant index analysis

After debonding, the zirconia surfaces were examined under stereomicroscope (Olympus SZ40-LG-PS2, Japan) focusing at (× 30) magnification. The Adhesive Remnant Index (ARI) scoring system developed by Årtun and Bergland [40], was modified to suit zirconia specimens. Failure modes were classified as one of four categories depending on the amount of adhesive remaining on the zirconia surface. According to this 4-point scale; score 0, indicates absence of composite remnants on the zirconia surface, denoting that bond fracture occurred at the resin/zirconia interface; score 1, indicates that less than 50% of composite remaining on the zirconia surface, denoting that bond fracture occurred predominantly at the resin/zirconia interface; score 2, indicates that more than 50% of composite remaining on the zirconia surface, denoting that bond fracture occurred predominantly at the bracket/resin interface; score 3, indicates that the entire composite remained on the zirconia surface with a clear impression of the bracket base on the remaining composite, denoting that bond fracture occurred at the bracket/resin interface.

### Scanning electron microscope (SEM) analysis

One additional zirconia specimen was prepared from each group using the same protocol and a control sample free of any surface treatment were explored using SEM (Quorum Q150T ES, Quorum Technologies Ltd, United Kingdom). Photomicrographs of representative areas of the zirconia surfaces were attained at (×1000) magnification. The SEM images were used to examine the surface morphology of zirconia samples after different treatments and compare them with control sample.

### Statistical analysis

Data were analyzed using statistical analysis performed with SPSS 16 ^®^ (Statistical Package for Scientific Studies), while Microsoft Excel software was used for data handling and graphical presentation. Descriptive statistics, including the mean, standard deviation (SD), the minimum, and maximum SBS values (MPa) were calculated; 95% confidence intervals were also included. The data was explored for normality using Shapiro-Wilk test and Kolmogorov-Smirnov test, which revealed that variables were normally distributed allowing the use of parametric test. Accordingly, comparison between different groups was performed by one-way analysis of variance (ANOVA) test, followed by Tukey`s Post Hoc test for multiple comparisons. Lastly, failure modes were presented as frequency (n) and percentages. Simple comparisons between groups were performed by Fisher’s exact test. The significance level was set at *P* ≤ 0.05 for all tests.

## Results

### Shear bond strength values

**Fig. 4:**
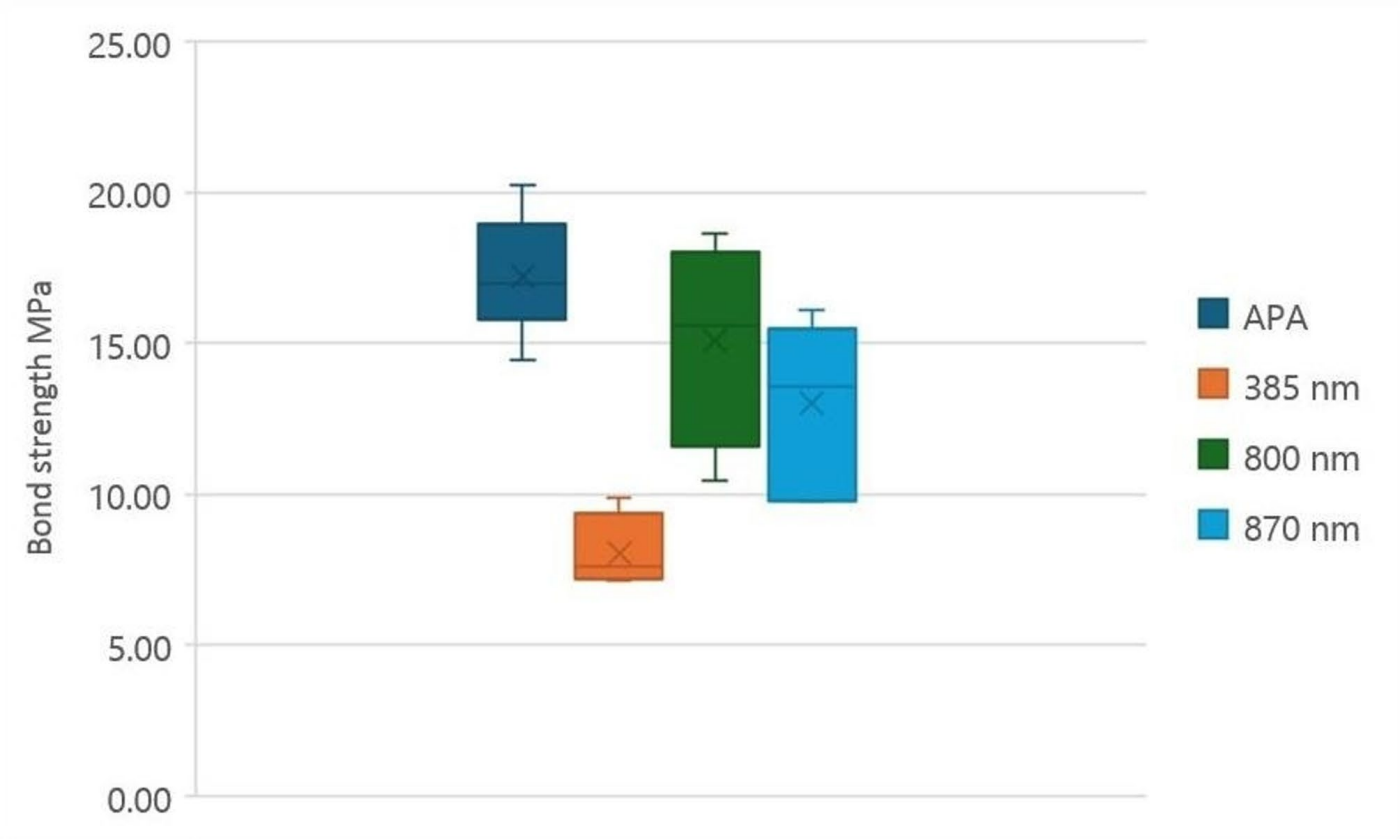
Boxplot representing descriptive statistical results of Shear bond strength (MPa) in all groups

The descriptive statistics of shear bond strength values (MPa) regarding all surface treatment groups are presented in Table 1 and Fig. 4.

**Table 1:** Descriptive results of shear bond strength (MPa) in all groups

Shear bond strength (MPa)	Minimum	Maximum	Mean	Standard Deviation	*P* value
APA	14.43	20.22	17.23	2.02	0.0001*
FS 385 nm	7.15	9.90	8.07	1.26	
FS 800 nm	10.47	18.65	15.07	3.42	
FS 870 nm	9.77	16.09	13.01	2.74	

ANOVA test revealed a highly significant difference among the groups (*P* = 0.0001), indicating that the different tested methods of surface treatment had a meaningful effect on SBS. Comparison between the testing groups revealed that the APA group recorded significantly the highest SBS (17.23 ± 2.02 MPa), followed by the FS 800 nm group (15.07 ± 3.42 MPa), then the FS 870 nm group (13.01 ± 2.74 MPa), while the FS 385 nm group showed significantly the lowest SBS (8.07 ± 1.26 MPa) with *P* = 0.0001.

Pairwise comparison between different groups was performed by using Tukey`s Post Hoc test. Results revealed that there was a significant difference between FS 385 nm vs. APA (9.16 as *P* < 0.0001), FS 385 nm vs. FS 800 nm (7 as *P* < 0.0001), FS 385 nm vs. FS 870 nm (4.94 as *P* < 0.0001), and FS 870 nm vs. APA (4.22 as *P* < 0.0001). However, there was an insignificant difference between FS 800 nm vs. FS 870 nm (2.06 as *P* = 0.11), and FS 800 nm vs. APA (2.16 as *P* = 0.09).

### Adhesive remnant index analysis

**Fig. 5:**
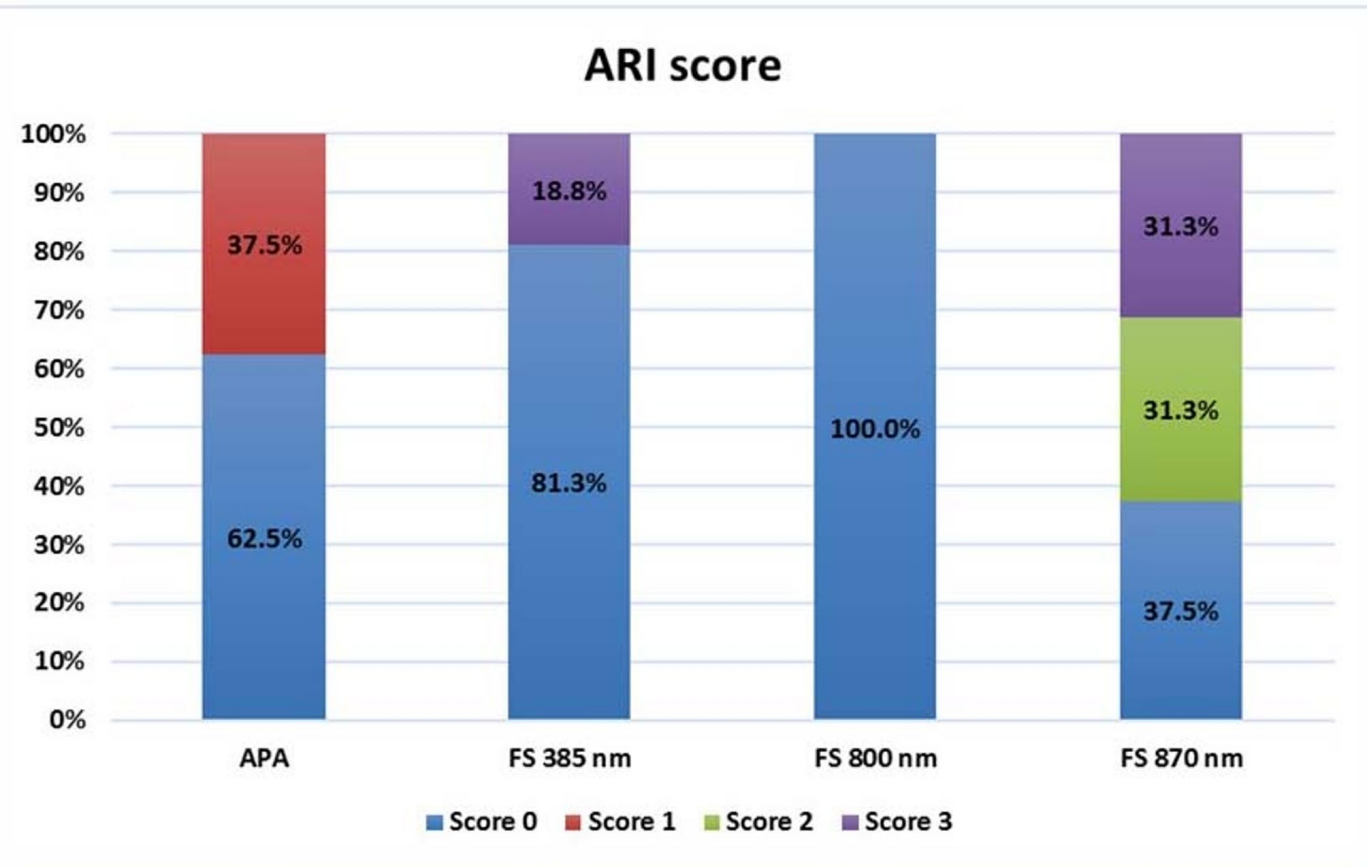
Stacked bar chart representing ARI scores distribution among APA, FS 385 nm, FS 800 nm, and FS 870 nm groups

**Fig. 6:**
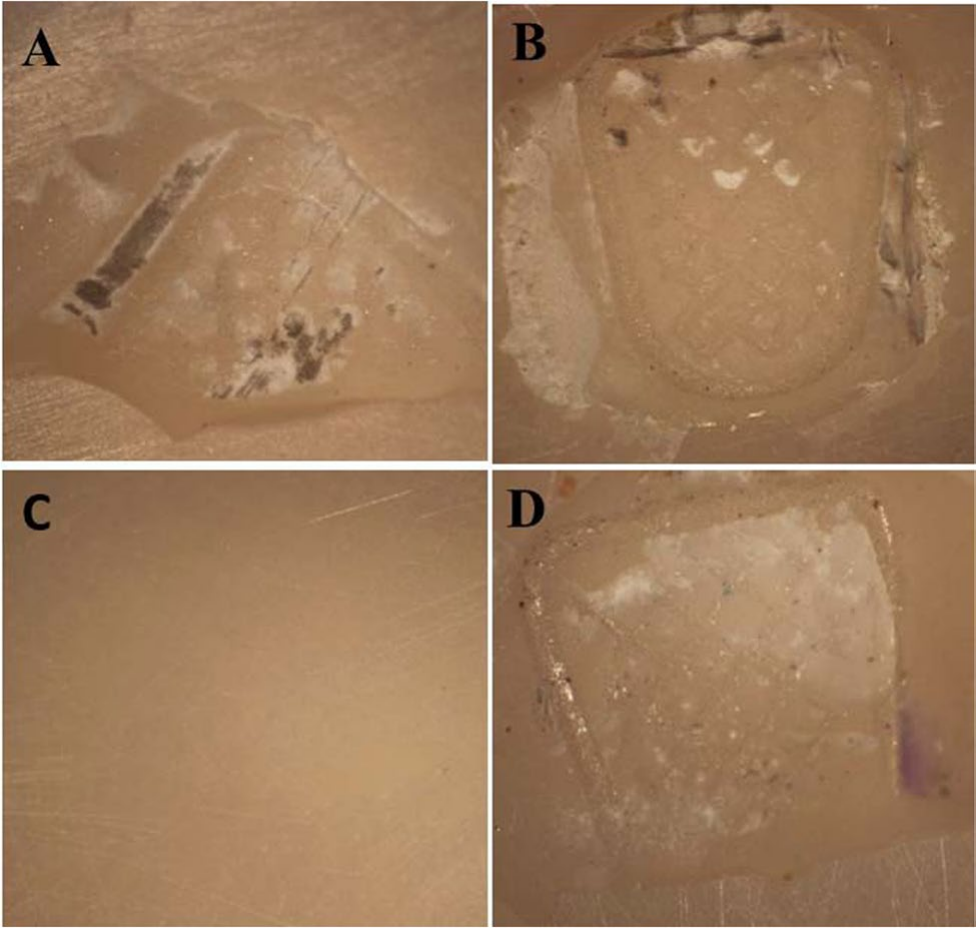
Representatives of stereomicroscope images. **A** ARI score 1 for an APA sample, (**B**) ARI score 3 for a FS 385 nm sample, (**C**) ARI score 0 for a FS 800 nm sample, and (**D**) ARI score 2 for a FS 870 nm sample

The ARI scores obtained after debonding for all the groups are presented in Fig. 5 (n and %). Representatives of stereomicroscope images are presented in Fig. 6. Results revealed that ARI score of 0 was predominant in all groups.

Comparison between groups revealed that there was a significant difference between them (*P* = 0.0001). Regarding the conventional APA method, 62.5% of the samples scored 0, while 37.5% scored 1. The FS 385 nm showed 81.3% scored 0, and 18.8% scored 3. All FS 800 nm samples (100%) scoring 0. A combination of scores 0, 2, and 3 was observed in FS 870 nm group with the following percentages, 37.5%, 31.3%, and 31.3%, respectively.

### SEM analysis of surface morphology

**Fig. 7:**
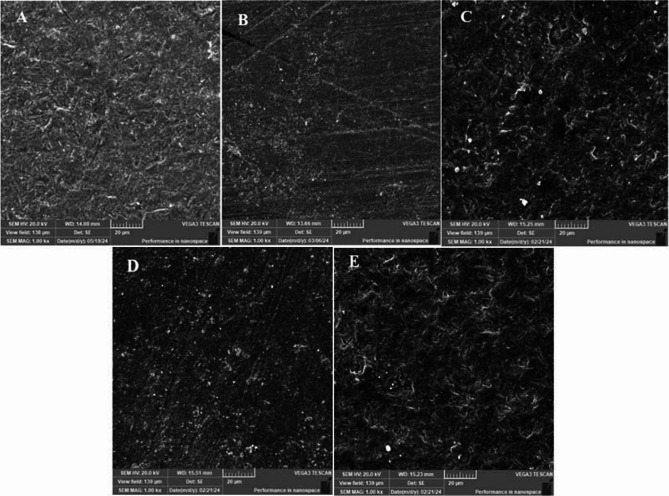
The SEM micrographs of zirconia ceramic surfaces after treatments at ×1000 magnification (**A**) No treatment; (**B**) APA; (**C**), (**D**), and (**E**) FS laser irradiation at different excitation wavelengths of 385 nm, 800 nm, and 870 nm, respectively

Differences in surface morphology were observed in SEM images of the specimens at × 1000, as shown in Fig. 7. The SEM image of the untreated surface (Fig. 7A) appeared relatively smooth when compared to the APA (Fig. 7B) and laser-treated specimens at different wavelengths of 385 nm, 800 nm, and 870 nm (Fig. 7C, D and E).

At ×1000 magnification, the untreated zirconia surface (Fig. 7A) exhibited a smooth surface except for some traces derived from the polishing procedure, lacking the microstructural features typically associated with enhanced bond strength. In contrast, airborne-particle abrasion (APA) specimen (Fig. 7B) revealed an irregular, roughened surface with distinct microretentive patterns, indicative of improved potential for micromechanical interlocking. Wavelength-dependent alterations in surface morphology were evident in the SEM examination of FS laser-treated zirconia. The FS 385 nm specimen (Fig. 7C) exhibited non-roughened surface with smooth texture and minimal grooves similar to the untreated group which could explain the significantly lower SBS achieved with this wavelength. The FS 800 nm image (Fig. 7D) displayed distinct micro-grooves and roughened textures, comparable to those found in APA. Those morphological changes might favor micromechanical retention and bonding potential. The FS 870 nm setting (Fig. 7E) displayed a moderately roughened surface with fewer pits and grooves compared to APA and FS 800 nm, though still providing textural changes favorable for bonding.

## Discussion

In orthodontics, bonding brackets to different surfaces including enamel and various restorations in an important procedure in everyday practice to ensure efficient force delivery through bracket attachments. Although several surface treatment techniques have been proposed for conditioning zirconia surfaces to improve shear bond strength of orthodontic brackets to zirconia, still a standardized conditioning protocol was not yet achieved [11, 12, 16, 21].

The aim of this study is to evaluate the shear bond strength (SBS) of metal brackets to multilayer zirconia following femtosecond laser surface treatments with different wavelengths.

The null hypothesis was rejected since the results of this study showed significant differences between different femtosecond laser wavelength settings used in the testing groups.

Lower incisors brackets were selected to conduct the study since these brackets have flatter bases than other teeth brackets, thus adapting better to the zirconia surfaces. The universal bond (Tetric N-Bond Universal, Ivoclar, Vivadent) was utilized in our study to simplify bonding procedure as recommended by Pédemay et al., and Hu Y et al. [10, 41]. They stated that bonding agents that contain 10-methacryloyloxy-decyl-dihydrogen-phosphate (10-MDP) greatly improves bonding to zirconia ceramiics. It has been suggested that the 10-MDP molecule bonds chemically to the zirconia surface through its phosphate functional group, while its methacrylate group offers a reactive site for bonding with the resin cement, thereby enhancing bond strength and durability. However, Lee et al. recommended the use of Porcelain primer (silane), instead of Zirconia primer (MDP monomer) for bonding metal brackets to glazed zirconia surfaces with resin cement. It has been suggested that glazed surfaces often contain silica along with small amounts of various metal oxides. Consequently, silane agents can bond to these surfaces through hydrophilic labile silanol groups (-Si-OH), which bond chemically with the hydroxyl (-OH) groups present on the silica surface, forming strong covalent siloxane bonds (-Si–O–Si-) [42].

During orthodontic treatment, an optimal bracket bond strength ranging from 5 to 10 MPa is required to deliver orthodontic movement forces and at the same time resists dislodgement by occlusal chewing loads [31, 43]. Reynolds et al. advocated that the minimum SBS required in orthodontic treatment is between 5.9 and 7.8 MPa [44]. On the other hand, excessive adhesion should be avoided as it increases the risk of cracking and fracture of the bonding surface, enamel or restoration during debonding; some authors have reported that the maximum bond strength should be 20 MPa [45], while others reported values between 40 and 50 MPa [46]. The results of our study showed that all the tested surface treatment protocols produced acceptable shear bond strength within the range of 8.07–17.23 MPa.

In our study, Hydrofluoric (HF) acid etching was not selected among the tested zirconia conditioning techniques as many previous studies confirmed that it is not effective with zirconia ceramics owing to their polycrystalline nature and the absence of Silica phase [10, 12, 29].

Airborne-particle abrasion (APA) was chosen as a traditional gold standard reference technique for comparison to femtosecond laser as many previous studies reported the high effectiveness of this protocol owing to the production of irregularities and shallow pits in the zirconia surface [16, 47]. In our study, APA group significantly increased the SBS and exhibited the highest mean (17.23 ± 2.02 MPa). This finding is consistent with other studies as reported by Ahmed, T. and N. Fareen in their systematic review [48]. Gomes et al. also found similar results of SBS with APA scoring 17.57 ± 5.27 MPa, and 20.18 ± 4.42 MPa for different Al_2_O_3_-size (25- µm and 50- µm, respectively [16]. Çevik et al. reported comparable outcomes for bonding to lithium disilicate and feldspathic ceramics, claiming that surface conditioning with sandblasting and grinding with a diamond bur achieved higher shear bond strength (SBS) values than chemical etching with hydrofluoric acid, phosphoric acid, and Nd-YAG laser irradiation. Given that the bonding mechanism between composite resin and ceramic is predominantly micromechanical, and the great ability of sandblasting to effectively remove the glazed layer, which otherwise has a detrimental effect on bond strength [49]. Hu Y et al. reported similar SBS values for metal brackets bonded to zirconia ceramics following sandblasting and the use of specific bonding agents containing 10-MDP monomer and adhesives free from 2-hydroxyethyl methacrylate (HEMA-free adhesive). However, in general the shear bond strength (SBS) values of ceramic brackets were lower than those of metal brackets after both 24 h of water storage (*P* >0.05) and after thermocycling [41].

In the current study, an ultrashort pulsed laser (femtosecond laser), has been used as a relatively innovative surface treatment technology capable of precisely producing microgrooves which can enhance bond strength without causing surface damage. A fully tunable femtosecond laser system was utilized to investigate the impact of different wavelengths on the shear bond strength of metal brackets to multilayer zirconia. To our knowledge, no previous studies explored this parameter. Most previous studies used fixed FS laser wavelength with a value of 800 nm while changing other parameters [29, 33–35]. Few studies used other wavelength values. Alsarani et al. irradiated their specimens with femtosecond laser beam set at wavelength 1026 nm, and yielded similar results of SBS 12.90 ± 2.90 MPa, 13.01 ± 2.21 MPa, and 17.57 ± 3.01 MPa for different surface patterns [25].

The investigation of our study highlighted that the bond strength of zirconia to metal brackets varies significantly depending on the surface treatment applied. Among the groups studied, the APA group exhibited the highest mean shear bond strength (17.23 ± 2.02 MPa), with no significant difference when compared to FS 800 (mean difference of −2.16 MPa, *P* = 0.09), and FS 870 nm (mean difference of 2.06 MPa, *P* = 0.11). The FS 385 nm group recorded the lowest SBS (8.07 ± 1.26 MPa). The current study aimed to confirm that the 800 nm wavelength was the most effective wavelength for enhancing SBS, a finding that was supported by Yavuz et al. [30].

Regarding the surface topographic examination, scanning electron microscope imaging revealed considerable quality changes in zirconia surface after femtosecond laser irradiation at 800 nm and 870 nm wavelengths. It is well recognized that the mechanical bonding relies immensely on the microstructure and surface texture of the zirconia Surface. APA treatment showed increased surface roughness and mechanical interlocking, providing a more substantial substrate for the adhesive which is more likely the cause for significant enhancement of the bond strength. The FS laser 800 nm treatment and 870 nm treatments showed distinct irregularities and pits comparable to those created by APA, which resulted in considerable improvement in bond strength. The lower performance of the 385 nm treatment might be attributed to insufficient alteration of the zirconia surface at this wavelength, leading to suboptimal bonding conditions.

The laser wavelength influence used to modify the zirconia surface may have a significant effect on its morphological properties. Studying the effect of three wavelengths of 385 nm, 800 nm, and 870 nm on the zirconia surface treatment, the infrared region is better than the ultraviolet region due to the IR high penetration depth of the sample [50]. Different wavelengths have great impact on the Zirconia surface treatment, with 800 nm being the most effective wavelength. This might be attributed to difference in the photon energy and number of photons incident on the zirconia surface. FS 800 nm revealed photon energy value of 1.55 eV, and the number of photons was 15.1 × 109, whereas FS 870 nm presented lower photon energy value of 1.42 eV and the number of photons was 16.5 × 109. Several studies have demonstrated the effectiveness of using an 800 nm Fs laser to roughen the zirconia surfaces without causing phase transformation [34, 51–54].

During bracket debonding, our results revealed that most testing groups demonstrated low ARI scores of 0 & 1, indicating that no or less than 50% of adhesive remained on zirconia surface. This is a favorable finding because it ensures that no cohesion breakdown occurs inside zirconia restoration and it also limits the risk of scratching zirconia surface by allowing easier removal of adhesive remnants [10]. Our results are consistent with the findings of existing studies [31]. Despite the enhanced shear bond strength values obtained from Ti: Sapphire laser in our study, a low ARI score was observed which contrast the findings of studies which denoted the occurrence of types 2,3, and 4 bond failure in femtosecond laser groups with and without APA [32, 33]. This might be attributed to the finding reported by Zachrisson which stated that the possibility of occurrence of ceramic breakdown during dedonding brackets was infrequent or absent in general regardless of the bonding method used previously [54]. Another interpretation of this apparent discrepancy might be due to the difference between the forces delivered from shear testing in the laboratory and the clinical debonding techniques [37, 55, 56].

According to the current findings, zirconia surface modification to improve bonding is more successful when done at infrared wavelengths (800 and 870 nm) than at UV wavelengths (385 nm). Variations in laser penetration and interaction with zirconia may be the cause of this. In particular, the wavelength of 800 nm might offer the best combination of photon energy and penetration depth to modify the surface sufficiently without causing microcracking or heat damage.

The limitation of the present study was using static shear bond strength testing machine which does not completely simulate the intra-oral forces falling on the brackets, so further studies are needed to implement cyclic shear bond strength testing to mimic the oral environment. The absence of aging simulation such as thermocycling test to determine the effect of thermal changes on the durability of bonding also added to the study limitation. This was attributed to the main goal of our study design which focused on determining the immediate bond strength which is also important in orthodontics during wire ligation in the brackets to deliver instantaneous force after bonding and to limit the parameters that might affect bond strength. Exploring other wavelengths as well as testing other laser parameters such as output power, pulse rate, laser patterns and angles are highly recommended in future studies to discover new protocols that maximize SBS while preserving zirconia’s structural integrity.

## Conclusions

Considering the limitations of this study, femtosecond (FS) laser treatments could be recommended as a promising alternative zirconia surface conditioning method to improve bonding of metal brackets to multilayer zirconia while minimizing the risks of surface and structural damage associated with abrasive techniques. The effectiveness of this technique was shown to be highly dependent on wavelength, with the near-infrared wavelengths (800 nm and 870 nm) significantly outperforming the ultraviolet wavelength (385 nm).

## Data Availability

The data is available with the corresponding author and available by reason of request.

## Abbreviations

FS: Femtosecond laser
APA: Airborne- particle abrasion
SBS: Shear bond strength
SEM: Scanning electron microscope
ARI: Adhesive remnant index
10-MDP: 10-methacryloyloxydecyl dihydrogen phosphate
HEMA: 2-hydroxyethyl methacrylate
HF: Hydrofluoric acid
MPa: Megapascal
nm: Nanometer
Zr: Zirconia
